# Novel four-exon deletion in ryanodine receptor gene (*RYR2*) associated with mixed electric and structural cardiac phenotype

**DOI:** 10.1093/europace/euaf189

**Published:** 2025-08-26

**Authors:** Tereza Stribna, Petra Peldova, Tatiana Vosecka, Tomas Roubicek, Milos Kubanek, Robert Cihak, Petr Peichl, Josef Kautzner, Milan Macek, Alice Krebsova

**Affiliations:** Department of Biology and Medical Genetics, Second Faculty of Medicine, Charles University and Motol University Hospital, Prague, Czech Republic; Department of Biology and Medical Genetics, Second Faculty of Medicine, Charles University and Motol University Hospital, Prague, Czech Republic; Department of Biology and Medical Genetics, Second Faculty of Medicine, Charles University and Motol University Hospital, Prague, Czech Republic; Department for Cardiology, Hospital Liberec, Liberec, Czech Republic; Faculty of Health Studies, Technical University Liberec, Liberec, Czech Republic; Department of Cardiology, Center for Inherited Cardiovascular Diseases, IKEM, Prague, Czech Republic; Department of Cardiology, Center for Inherited Cardiovascular Diseases, IKEM, Prague, Czech Republic; Department of Cardiology, Center for Inherited Cardiovascular Diseases, IKEM, Prague, Czech Republic; Department of Cardiology, Center for Inherited Cardiovascular Diseases, IKEM, Prague, Czech Republic; Department of Biology and Medical Genetics, Second Faculty of Medicine, Charles University and Motol University Hospital, Prague, Czech Republic; Department of Cardiology, Center for Inherited Cardiovascular Diseases, IKEM, Prague, Czech Republic

**Keywords:** *RYR2* deletion, CNV analysis, LV hypertrabecularization, PVC from aortic root, Radiofrequency ablation, E3DS

## Introduction

Ryanodine receptor 2 gene codes for a Ca^2+^ channel involved in excitation–contraction coupling in the cardiac muscle.^1,2^ Missense mutations in *RYR2* cause catecholaminergic polymorphic ventricular tachycardia (CPVT),^3,4^ while an exon 3 deletion has been reported in patients with variable clinical manifestations including supraventricular arrhythmias, conduction disorders, ventricular arrhythmias and left ventricular (LV) hypertrabecularization.^5–9^ This syndrome, also called exon 3 deletion syndrome (E3DS), is now considered a distinct diagnostic entity among RYR2-ryanodinopathies.^10^

**Figure 1:**
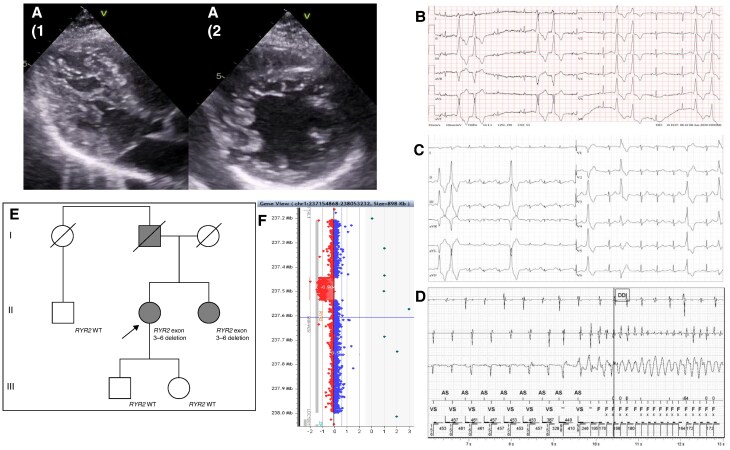
(*A*) Hypertrabecularization in echocardiography PLAX (*A1*) and PSAX (*A2*). (*B*) ECG with PVC from aortic root in proband's sister. (*C*) Documented bidirectional PVS during exertion in proband. (*D*) Documented VF in proband's ICD. (*E*) Three-generational pedigree with familial segregation of the RYR2 deletion. The affected individuals are marked with full circles/squares. Individuals I. generation died prior to genetic testing, no DNA was available. (*F*) Graphical representation of the RYR2 deletion (NM_001035) by aCGH showed a partial deletion in the 1q43 region concerning RYR2 exons 3–6.

Here, we report a unique case of a four-exon deletion (3, 4, 5, and 6) in the *RYR2* gene in a Czech family of three affected individuals with mixed electric and structural cardiac phenotype (*Figure 1*).

Proband manifested with exertion-related syncope at the age of 45 years. Spontaneously terminating atrial fibrillation (AF) and later documented sustained ventricular tachycardia together with LV hypertrabecularization with transitory reduction of left ventricular ejection fraction (LVEF 30%) and no late gadolinium enhancement (LGE) led to ICD implantation in secondary prevention shortly after initial diagnosis. Pulmonary vein isolation was performed reaching no documented AF event during the 6-year follow-up. Adequate ICD therapy for ventricular fibrillation (VF) resulting from sinus rhythm during dancing at the ball was observed (*Figure 1*). On rest and exercise electrocardiograms (ECGs), frequent premature ventricular contractions (PVCs) from the left aortic cusp as well as bidirectional PVCs upon exertion were documented (*Figure 1*).

Family cascade screening led to the identification of LV hypertrabecularization with reduced systolic function (LVEF 35%) and no LGE in cardiac magnetic resonance in proband’s younger sister. She was implanted with an ICD in primary prevention. Resting and exercise ECGs showed frequent PVCs from the right aortic cusp. Catheter ablation of the right aortic cusp resulted in no recurrence at the 3 and 9 months follow-up. Additionally, she has a bicuspid aortic valve (BAV) without relevant dysfunction and discrete, non-progressive aortic dilation (max. 37 mm–22 mm/m^2^) without other signs of syndromic aortic diseases.

The proband’s father died at the age of 68 due to terminal heart failure accompanied by recurrent malignant ventricular arrhythmias. He was diagnosed at the age of 50 with a combination of ischaemic cardiomyopathy and pronounced LV hypertrabecularization with reduced LVEF (30%) and without valvular or aortic disease. On resting ECGs, frequent PVCs from the right aortic cusp, identical to his living daughter, were documented. He died before molecular genetic analysis was available.

The proband’s mother died from intracranial haemorrhage at the age of 66 years and had no cardiac disease or arrhythmia during her lifetime.

The proband’s offspring have no arrhythmias or LV hypertrabecularization. Her son has BAV with mild aortic dilatation. The paternal cousin of the proband has no cardiological findings, nor did his deceased mother.

## Methods

The proband and her affected sister were examined by massively parallel sequencing (MPS, Sophia Genetics, Switzerland) using a customized panel of 100 cardiovascular genes and the Clinical Exome Solution (v3.1, 4727 genes), respectively. Bioinformatic analysis was performed using a pipeline in the SOPHiA DDM™ platform (Sophia Genetics). To verify copy number variation (CNV) findings, array comparative genomic hybridization (aCGH, SurePrint G3 Custom CGH+SNP 4x180K, Agilent, USA) and multiplex ligation probe amplification (MLPA, Probemix P168, MRC Holland; The Netherlands), were performed in both of them.

MLPA analysis was used for segregation analysis in other living relatives (two healthy offspring of the proband and a healthy paternal cousin (*Figure 1*)).

## Results

A CNV involving deletion of four consecutive exons^3–6^ in the *RYR2* gene was identified by MPS in the proband and her sister. No other variants (VUS/LP/P) were found in genes associated with development of cardiomyopathy, arrhythmias, and familial BAV in both of them. The aCGH method confirmed deletion of exons 3–6 in the 1q43 region of at least 92 kb (max. 92.5 kb), while MLPA containing only the probe for exon 3 allowed to confirm this deletion at least in its partial extent.

MLPA analysis in tested healthy living relatives excluded the pathogenic variant (*Figure 1*).

## Discussion

To our knowledge, this is the first case report that describes a unique, pathogenic heterozygous deletion spanning exons 3, 4, 5, and 6 of the *RYR2* gene. This finding underscores the necessity of CNV analysis within MPS. Regardless of kit used (custom panel or clinical exome), a four-exon deletion was detected with high accuracy in each case, indicating the reliability of used MPS methods.

MLPA is a reliable, time-, and cost-effective method for CNV detection; however, no commercial kit is currently available for the full range of the *RYR2* gene. The manufacturer could consider expanding the current product offering.

Cardiac manifestations in the reported sisters and their father (from whom they presumably inherited the deletion) are largely consistent with previous reports in patients with E3DS. However, frequent monomorphic PVCs originating in the region of the aortic root were on top of CPVT typical arrhythmia documented.

Our genetic findings could lead to a revision of the previously proposed term E3DS^10^ to a more general term that reflects the fact that RYR2 deletions may involve larger regions of the gene beyond exon 3, resulting in mixed structural and arrhythmic phenotypes.

Catheter ablation of AF and PVCs originating from the aortic root was effective and could be considered for such patients. Conversely, our observations may imply the need for a detailed clinical and/or genetic evaluation for LV hypertrabecularization and the risk of life-threatening arrhythmias in patients with frequent PVCs originating from the aortic root. Further studies and international registries of similar cases could improve our understanding.

## Data Availability

Data available on request.
